# ONLINE vs. FACE-TO-FACE group coaching to promote teachers mental health: an exploratory field study in German teachers

**DOI:** 10.3389/fdgth.2025.1479524

**Published:** 2025-02-05

**Authors:** Sarah S. Lütke Lanfer, Ruth Pfeifer, Yannik Rieder, Alexander Wünsch, Matthias Braeunig, Claas Lahmann

**Affiliations:** ^1^ Department for Psychosomatic Medicine and Psychotherapy, Medical Center—University of Freiburg, Faculty of Medicine, University of Freiburg, Freiburg, Germany; ^2^ Department of Medical Oncology, Inselspital, Bern University Hospital, University of Bern, Bern, Switzerland

**Keywords:** online, teacher, mental health, prevention, face-to-face, balint technique

## Abstract

**Introduction:**

Online formats provide promising and low-threshold options for mental health coaching. However, research on online mental health interventions compared to traditional face-to-face interventions remains scarce. In the present study, the established prevention tool “*Teacher Group-coaching Program*” (TGP) was applied in both the original face-to-face setting as well as online. TGP focuses on promoting mental health in teachers by strengthening relational skills using the Balint group technique. This technique roots back to a psychoanalytic approach to explore the emotional aspects of (stress inducing) professional relationships. The current study aimed at comparing the satisfaction with and effectiveness of TGP between both settings.

**Method:**

The sample consisted of 104 teachers who voluntarily chose between face-to-face (*n* = 51) and online (*n* = 53) setting. In a pre-posttest design, participants completed questionnaires before and after the intervention. Additionally participant's satisfaction with the program was assessed during and after TGP.

**Results:**

Intervention effects did not differ significantly in terms of mental health, general life satisfaction and emotional distancing between TGP online and face-to-face. In line with previous research, there was a pre-posttest improvement for mental distress and the ability to distance oneself for both groups, which did not differ significantly between face-to-face and online setting. Satisfaction with the program was rated high in both settings, suggesting similar acceptance.

**Discussion:**

Although, the absence of an effect is not the evidence of equality of the groups, the present study highlights the potential of online admissions of mental health interventions as possible alternatives and additions to traditional face-to-face programs, especially when in-person meetings are not feasible. Specifically, it shows evidence that the Balint group technique can also be applied successfully by trained experts in the online setting.

## 1 Introduction

Digitization is a global issue that has become an integral part of our daily lives. With the outbreak of the COVID-19 pandemic in spring 2020, its importance has been reinforced in almost all areas of life (1, 2). A growing need for support services, particularly in the field of mental health (3) and an increased prevalence of mental diseases [e.g., (4, 5)] made it also necessary for coaches and clinical therapists to search for digital ways to continue reaching people (6–8). Therefore, online coaching to maintain and improve mental health has become even more popular [e.g., (9–13)]. The pandemic also fostered the successful transformation of psychotherapeutic treatment to a digital setting (14, 15). Several studies have demonstrated the general effectiveness of various online-based psychotherapy approaches for depressive symptoms [e.g., (16–18)], anxiety disorders [e.g., (19, 20)] as well as for various other mental disorders [e.g., (21–23)]. A systematic review and meta-analysis of early research in the context of the pandemic by Chi et al. (24) showed that online psychological interventions could effectively reduce COVID-19 induced depression, anxiety, and stress levels. Comparing online with face-to-face settings, Axelsson et al. (19) found in a randomized controlled trial that cognitive behavioral therapy produces equivalent results in improving mental health in both settings, as shown in a pre-pandemic meta-analysis (21). Other research has shown that workplace-related online coaching to improve mental health can also be successful (25–27) and increase productivity (25). A meta-analysis of Jones, Woods, & Guillaume (28) found no significant differences in organizational outcomes (cognitive, skill-based and affective outcome criteria) between online and face-to-face coaching in the workplace. In contrast, to our knowledge, no research exists so far that directly compared online and face-to-face coaching aiming to improve employees' mental health in a single study. The present study is directed at this research gap by investigating the acceptance and effectiveness of an established work-related coaching program to promote teachers' mental health in both the online and the face-to-face setting.

In spite of the importance of education for society and its' development (29) alarming reports from all over the world are drawing attention to the state of health in teachers [e.g., (30–34)]. Teachers' mental health has been worldwide a recurring topic for decades (35–40) and is often linked with the phenomenon of “burnout” and early retirement (41–46). A large body of research shows that teachers have higher rates of mental and psychosomatic disorders than other professions (47–51). Essential reasons for teachers' distress include high workload, time pressure, low salaries, insufficient breaks during workdays, and too much administrative work [e.g., (52, 53)]. In addition, negative experiences in relationships with students, parents and colleagues have been identified as one of the key factors for teachers' mental health problems [e.g., (35, 54–60)]. The work-related measures (e.g., hygienic, contact rules) applied during the pandemic intensified the stressful work environment for teachers by requiring constant adaption to changes in politics and school regulations. Unsurprising, an even higher level of stress symptoms and mental disorders in teachers has been reported since the COVID-19 outbreak [e.g., (32, 61–64)]. Thus, it is of vital importance to develop effective and accessible intervention programs to promote mental health of teachers.

Organizational interventions to improve teachers' mental health are targeting two main areas: First, modify the working environment to reduce the probability of mental health issues (system-level oriented) or, second, strengthen employee's abilities and resources to cope better with stress inducing work situations (behavior or individual-level oriented). As the teaching environment is very much influenced by politics and job inherent factors, focusing on the second area seems more promising. Studies show that successful teacher-student relationships strongly contribute to the quality of teaching (65), while dysfunctional teacher-student relationships are one of the most significant burdens on teacher health (35, 56, 58, 60). Therefore, the “*Teacher Group-coaching Program*” (TGP) (66) which is designed to strengthen resilience by focusing on the relational skills of teachers should be a good way to promote teachers' mental health. The intervention is conducted by trained psychotherapists or school psychologists and follows a standardized procedure which is built around the Balint group technique (67). Studies showed positive effects of TGP with respect to mental distress (68–70), burnout (71), effort-reward-imbalance (71), self-efficacy (72) and general work-related attitudes (68). Since 2012, a federal state government in Germany has therefore offered all public school teachers free participation in the coaching groups as a health and safety measure. Originally, TGP was designed to be a face-to-face group intervention and was exclusively conducted in this setting. However, due to the strict regulations during the pandemic period, groups had to be cancelled and there was a dangerous risk that an important source of support for teachers would disappear. Thus, TGP was carried out online as an add-on to meet high demand during the pandemic period. However, offering TGP in this new setting raises the question of whether the TGP online version differs from the original face-to-face setting in relation to acceptance and results in improving teachers' mental health. Specifically, we consider two aspects to answer the question: evaluation during the program and pre-post-comparison. First, we focus on how participants in the online group and face-to-face setting evaluate the program during participation (satisfaction). Second, training effects on teachers' mental health and work-related attitudes were compared between online- and face-to-face-setting in a pre-post-design (mental health, work-related attitudes).

## 2 Materials and methods

### 2.1 Study design and participants

The initial sample of this study included 860 German teachers, who participated in TGP in the academic years 2020/21, 2021/22, and 2022/23, respectively. Most of the participants were female (85%) and had no leadership role (85%), participants worked on average 14 years (+/−8,6 years) as teachers. The present study is part of a larger prevention study for teachers in public schools in southern Germany and used a longitudinal quasi-experimental pre-post-test design to assess the training effects of TGP on mental health and work-related attitudes. The response rate for T1 (pre-test) varied between 46% (academic year 2020/2021) and 60% (academic year 2022/2023).

Participants were eligible for the study if they indicated that they attended at least five of the six prescribed TGP sessions and submitted both the pre- and post-test questionnaires that could be matched by their self-generated code. Participation in the questionnaires and in TGP was voluntary and anonymous. Therefore, the drop-out rate resulting from non-attendance cannot be assessed. Informed consent was given by the participants allowing the use of the data for research purposes by completing the questionnaire. The ethics committee at Freiburg University, Germany approved TGP, as well as the applied questionnaire.

**Figure 1:**
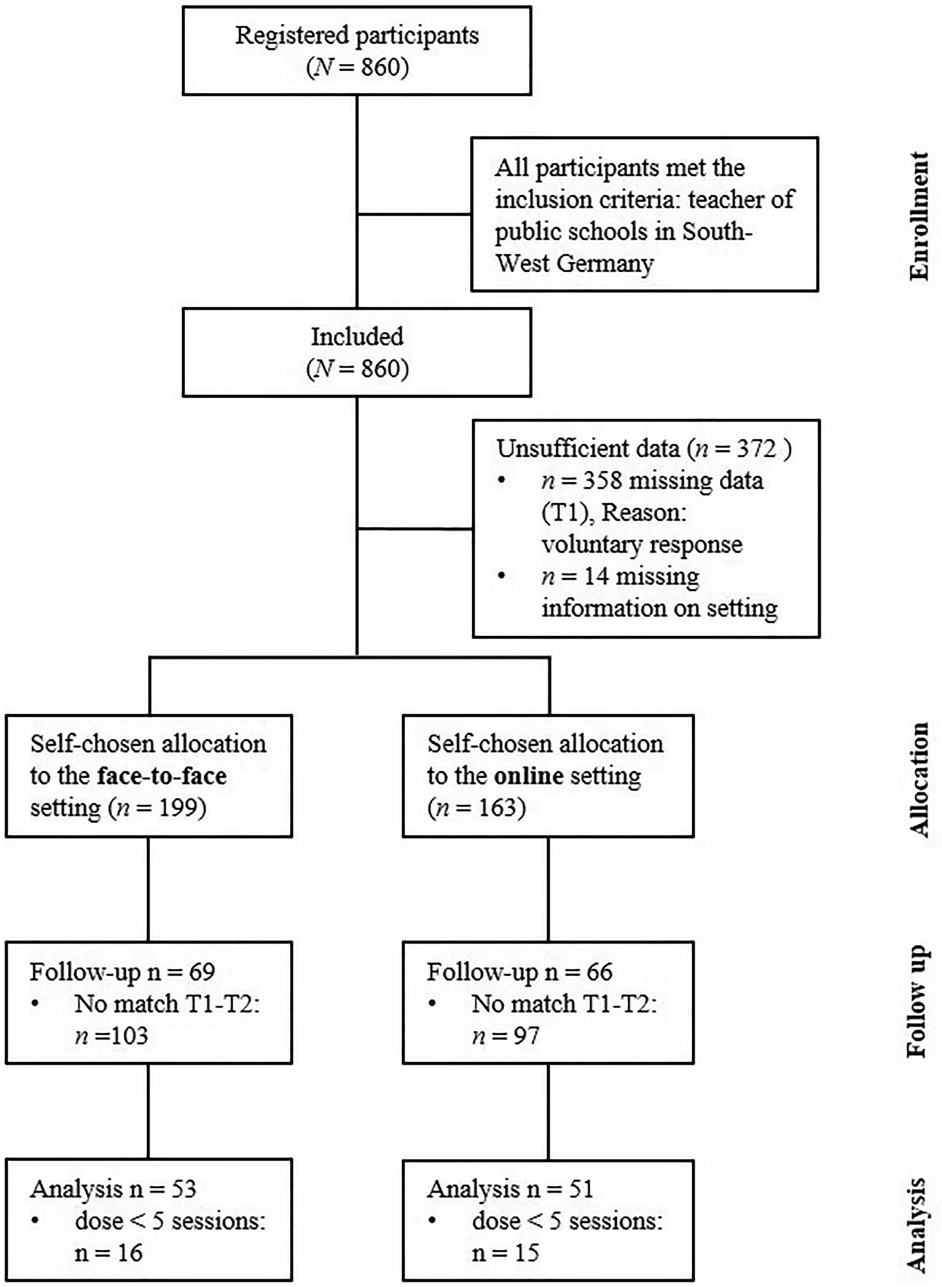
Flow diagram of group compositions. For group comparisons see Table 1 and Supplementary Table S1.

For the analyses of the time effects, 756 participants had to be excluded from the study, as they did not meet the inclusion criteria (missing pre- or post-test data, less than five sessions, no match, see Figure 1). The reasons behind the exclusion criteria are the following:

a. missing pre- or post-test data: As participation in the questionnaire was voluntarily, some participant only completed T1 while others only completed T2.
b. less than five sessions: In order to have the full dose of training, participants had to participate at least five of the six coaching sessions. This threshold was conducted from previous research.
c. no match: T1 and T2 could not be matched by an individual code which was set up by the participants.

Excluded participants who participated in T1 (pre-test) did not significantly differ concerning age, gender, teaching load, leadership role and school type from the study sample (see Supplementary Table S1).

Thus, the *final study sample* comprised 104 teachers of which 51 participants attended the face-to-face setting (face-to-face group) and 53 participated in the online setting (online group), respectively. Most of the participants were female (90.4%), older than 50 years of age (49%), had no leadership role (78.8%) and worked part time (72.5%). The two samples did not significantly differ in gender, teaching load, leadership role and school type (see Table 1). However, participants in the online group were younger (66% < 50 years) than in the face-to-face group (35% < 50 years). An *a priori* power analysis conducted using G*Power (Version 3.1) (73) determined that a sample size of 34 participants would be sufficient to achieve adequate statistical power (*β* = .80) for a repeated measures ANOVA with a within-between interaction, assuming a medium effect size (*f* = 0.25).

**Table 1:** Demographics, teaching and training related variables for the study sample.

	face-to-face group	online group	*χ*²-test *p*
*N*	51	53	
Demographics			
Gender			.163
female	44 (86.3%)	50 (94.3%)	
male	7 (13.7%)	3 (5.7%)	
Age (years)			.012
<35	2 (3.9%)	10 (18.9%)	
35–39	4 (7.8%)	5 (9.4%)	
40–44	3 (5.9%)	10 (18.9%)	
45–49	9 (17.6%)	10 (18.9%)	
50–54	18 (35.5%)	7 (13.2%)	
>55	15 (29.4%)	11 (20.8%)	
Teaching related variables			
Teaching load			.795
100%	19 (37.3%)	20 (37.7%)	
>75%	14 (27.5%)	13 (24.5%)	
50%–75%	17 (33.3%)	17 (32.1%)	
<50%	1 (2.0%)	3 (5.7%)	
Leadership role			.180
No	43 (84.3%)	39 (73.6%)	
Yes	8 (15.7%)	14 (26.4%)	
School type			.737
Basic elementary school (1st–4th year)	14 (27.5%)	11 (20.8%)	
Secondary school (5th–9th year)	1 (2.0%)	2 (3.8%)	
Secondary school (5th–10th year)	4 (7.8%)	6 (11.3%)	
High school (5th–13th year)	9 (17.6%)	14 (26.4%)	
Community school	3 (5.9%)	2 (3.8%)	
Vocational school	8 (15.7%)	10 (18.9%)	
Special schools for mentally or physically handicapped pupils	12 (23.5%)	8 (15.1%)	
Leadership responsibility			.180
No	43 (84.3%)	39 (73.6%)	
Yes	8 (15.7%)	14 (26.4%)	
Training related variables			
Repeated participation			.057
No	24 (47.1%)	32 (60.4%)	
Yes	27 (52.9%)	21 (39.6%)	
Academic year			.287
2020/21	10 (19.6%)	15 (28.3%)	
2021/22	13 (25.5%)	17 (32.1%)	
2022/23	28 (54.9%)	21 (39.6%)	

*N* = 104; frequency (percentage in group); *χ*²-test *p* between groups.

### 2.2 Procedure

At the beginning of each academic year, TGP was announced to all public schools via the newsletters of Center for School Quality and Teacher Education. Teachers who decided to participate registered online for the intervention and could choose a suitable TGP group themselves. The groups varied according to setting (face-to-face vs. online), date, place, moderator and participants (e.g., leadership role: yes vs. no). Thus, participants chose between online and face-to-face setting during their registration and were assigned to the face-to-face or online group setting accordingly (see Figure 1). Upon registration all participants were asked to complete a questionnaire comprising demographic data as well as questions concerning mental health and their work-related attitudes (pre-test, T1). The respective moderator invited teachers to their registered groups and joined the TGP group. Both, face-to-face and online groups received 6 × 130 min of group coaching (see Intervention). Participation in TGP was voluntary. Participants could discontinue TGP at any point in the process. During TGP, participants were asked to answer six questions concerning their satisfaction with the group process at the end of the third and sixth group sessions. Two weeks after the end of the intervention, participants were invited to take part in the post-test questionnaire (T2), which included the pre-test questions and additionally the satisfaction evaluation questions. In the face-to-face group setting, questionnaires concerning satisfaction with the program during the TGP were collected using the paper-pencil-method. All other surveys were collected via a cloud-based web application.

### 2.3 Intervention

The *teacher group-coaching program* (TGP, 66) aims to strengthen teachers' resilience and competence in relationship management. Moderators who are licensed psychological professionals (either psychotherapists or school psychologists) coach the groups. All moderators completed a TGP training course and, to maintain a high quality of service were offered regular participation in annual conferences and intervention sessions. Additionally, the project management offers individual supervision.

TGP uses a standardized manual and is structured into five modules. Separate coaching groups are offered for teachers depending on whether or not they have leadership roles. TGP takes place about once a month in six recurring sessions of 130 min each. Five of the six sessions start with psychoeducation in one of the following five topics: (1) basic knowledge of stress physiology and the effects on health parameters; (2) mental attitudes with a particular focus on authenticity and identification; (3) competence in handling relationships with students; (4) competence in handling relationships with parents; and (5) strengthening co-worker relationship and social support (see Supplementary Table S2). The modules are named accordingly. After psychoeducation, the Balint group technique (67) as the core principle of the coaching is applied, focusing on difficult interpersonal situations during the teachers working life. The primary aim of a Balint group is to improve the provider-recipient relationship by exploring the feelings, thoughts, and behaviors of professionals in response to their encounters. In each session, one participant shares a detailed story of difficult interactions with others, focusing on their emotional responses rather than on situational details. The group responds to this report and provides their thoughts, feelings, and experiences, which helps foster understanding and outside perspectives (74). The moderator as trained professional enables the group and each participant by asking questions that help solve the conflict and foster understanding of oneself and the situation. As a third element, a relaxation technique is applied which can be practiced in each session as well. The last session has no specific topic and can be structured by the moderator according to the groups need.

TGP is an established coaching program that has been shown effective in the face-to-face setting to improve teachers mental health in a RCT study: The randomized controlled trial could show that TGP had a positive effect on burnout symptoms, effort-reward imbalance, and mental health (70, 71). Consequently, the program is offered to all state-employed teachers in a state in southern Germany since 2012.

TGP in the *online setting* used the same standardized procedure as the face-to-face setting, but was conducted online using the BigBlueButton (BBB) video platform (BigBlueButton Inc., https://bbb.lehrer-coachinggruppen.de). The platform adheres to strict privacy regulations and is cleared by the data protection office of the Medical Centre, University of Freiburg. Moreover, participants are generally familiar with this platform as it was established by the Center for School Quality and Teacher Education as teaching tool in all public schools.

### 2.4 Instruments

#### 2.4.1 Satisfaction with TGP (formative evaluation)

Satisfaction with the coaching program was measured using six statements: “topics were relevant for me” (interesting topics), “intra-group communication was constructive” (intra-group communication), “I have learned something” (learning experience), “I feel relieved by today's session” (relieving), “moderation of the group was good” (quality of moderation), and “the group session was of personal value for me” (perceived personal value). The items were answered on a scale ranging from “not true at all” (0) to “exactly true” (5).

#### 2.4.2 Intervention effects

##### 2.4.2.1 Mental health

Participants' mental health was measured using the short version of the General Health Questionnaire (GHQ-12, 75). Twelve items must be answered on a four-point Likert scale from 0 to 3. This scoring strategy allows for a sum score ranging from 0 to 36. Higher scores indicate a higher likelihood of psychiatric distress or potential mental health issues. Several studies have shown that the GHQ-12 is a reliable measure of mental health in several populations with and without mental health issues [e.g., (76, 77)]. This study used the German version of the GHQ-12 which also demonstrates strong reliability and validity in teachers (78).

##### 2.4.2.2 Work-related attitudes

In addition, two subscales of the short version of the Work-Related Behavior and Experience Patterns Questionnaire [AVEM-44 (79)], were used: *General life satisfaction* and *ability to distance oneself.* The AVEM is a psychometric tool designed to evaluate behaviors and experiences that either promote health or pose a risk in coping with work demands. Each subscale is comprised of 4 items that were scored on a five-point Likert scale: “strongly disagree” (1) to “strongly agree” (5). Higher scores indicate a more pronounced tendency of the respective dimensions. Both scales show high internal consistency as evidenced by Cronbach's alpha values ranging from.87 –.88 for the ability to distance oneself and .81–.82 for general life satisfaction (79).

#### 2.4.3 Demographics

Data on gender, age, teaching load, leadership role, and school type were collected.

### 2.5 Data analyses

All analyses were conducted using IBM SPSS Statistics 29.0.2 (IBM Corp.; Armonk, NY, USA). Effect size was calculated using partial eta squared, categorizing the effect sizes as small (*η_p_*^2^ = 0.01), medium (*η_p_*^2^ = 0.06), and large (*η_p_*^2^ = 0.14).

## 3 Results

### 3.1 Satisfaction with TGP (formative evaluation)

Participants were asked to evaluate TGP twice during the group coaching process: at the end of sessions 3 and 6, respectively. The satisfaction with the following six areas was rated: interesting topics, intra-group communication, learning experience, relieving, quality of moderation, and perceived personal value. To investigate, whether there were differences between face-to-face and online groups in satisfaction, for each evaluation, a MANOVA with setting (face-to-face vs. online) as independent variable and evaluation questions as dependent variables was calculated. Due to the required anonymity during the group coaching process within-person comparison was not possible.

**Figure 2:**
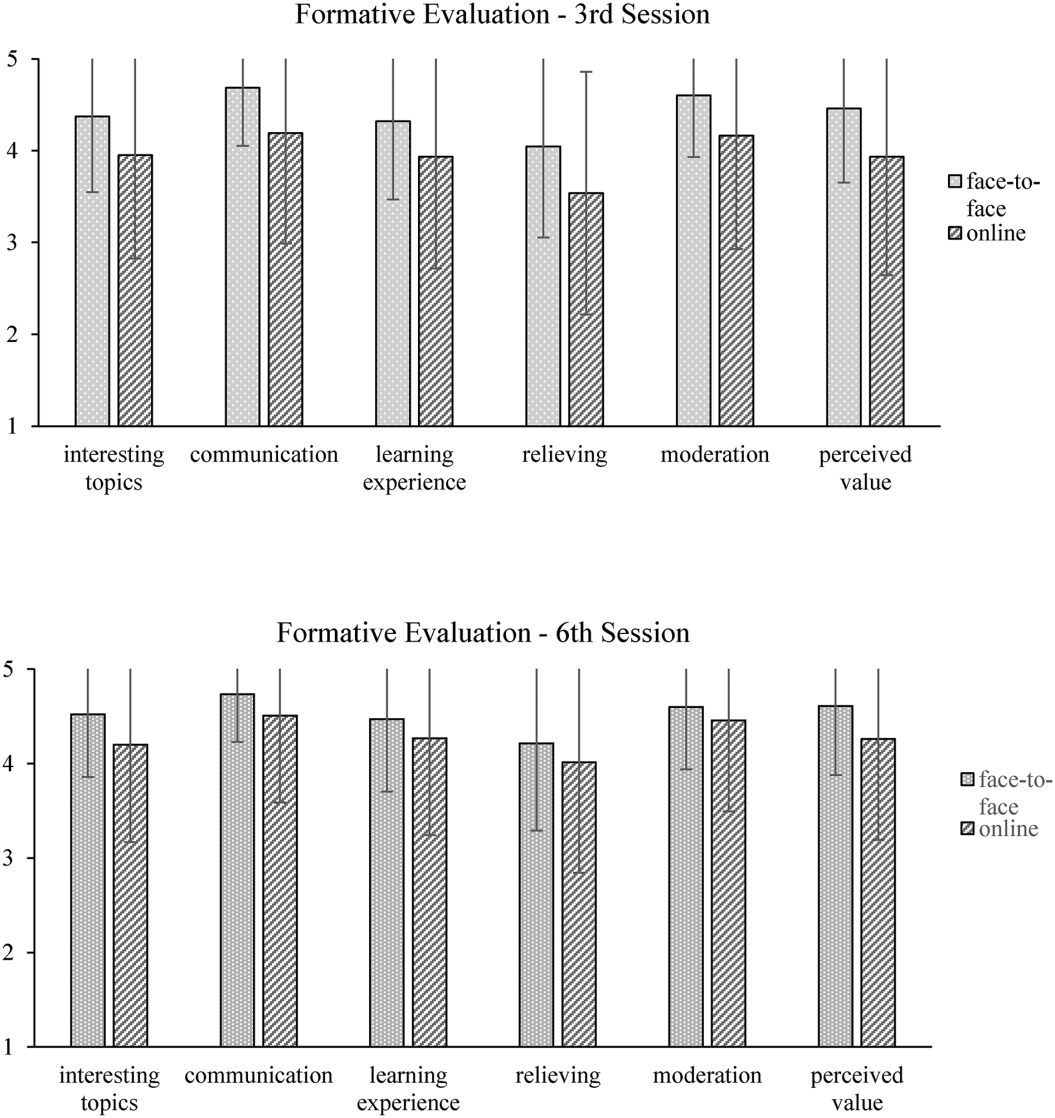
Formative evaluation in sessions 3 (*n* = 488) and 6 (*n* = 420) between online and face-to-face settings. Scale ranging from “not true at all” (0) to “exactly/very true” (5).

At the end of session 3, participants in the face-to-face setting rated satisfaction with all six areas greater than 4 (good). Participants in the online group rated all questions greater than 3.5. Overall, results showed a significant difference between the online and face-to-face group, *F*(6, 481) = 6.96, *p* < .001, *η_p_*^2^ = .08. *post-hoc* analyses revealed significant differences in all six items [*F*(1, 486) = 16.85–33.38, *p* < .001, *η_p_*^2^ = .03 −.06], showing that participants in the online group showed lower satisfaction than participants in the face-to-face group (see Figure 2).

At the end of session 6, the mean score for all six areas was above 4 (good) in both setting groups. Again, MANOVA results revealed significant differences between groups over all six items, *F*(6, 413) = 4.38, *p* < .001, *η_p_*^2^ = .06. *post-hoc* analyses showed that, in four areas, the face-to-face coaching group had significantly higher satisfaction scores than the online group: topics [*F*(1, 418) = 15.02, *p* < .001, *η_p_*^2^ = .04], intra-group communication [*F*(1, 418) = 10.76, *p* = .001, *η_p_*^2^ = .03], learning experience [*F*(1, 418) = 5.20, *p* < .05, *η_p_*^2^ = .01], perceived personal value [*F*(1, 418) = 15.46, *p* < .001, *η_p_*^2^ = .04], see Figure 2.

At T2 (two weeks after the last group session), teachers who participated in more than five of six sessions answered the same items to rate their experience with the group program. To investigate whether the online group evaluate the group program as positive as the face-to-face group after TGP, a MANCOVA with setting (face-to-face vs. online) as independent variable and evaluation questions as dependent variables was calculated. Gender, age, leadership role, teaching load and repetition of the coaching served as control variables. Results showed a tendency (*p* < .1) towards a significant main effect for setting, *F*(7, 96) = 1.94, *p* = .07, *η_p_*^2^ = .13. Exploring between-group differences, *post-hoc* analyses showed only significant differences between face-to-face and online group setting for moderation, *F*(1, 96) = 4.18, *p* < .05, *η_p_*^2^ = .04. Participants in the online setting gave higher ratings for their moderator. No control variable showed significant influence.

### 3.2 Effects of TGP on mental health and work-related attitudes

**Figure 3:**
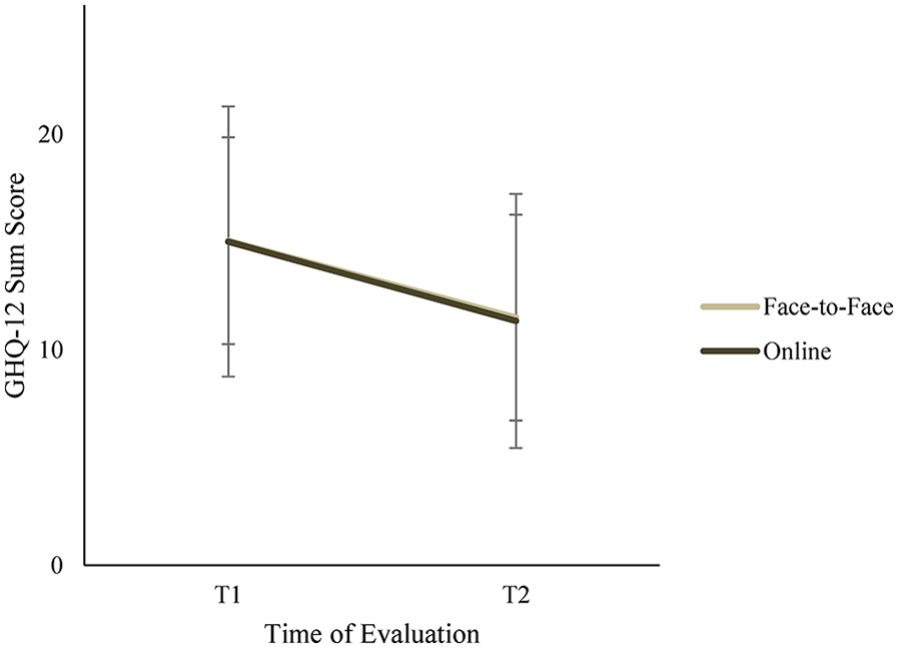
Mental health measured by the GHQ-12 before (T1) and after (T2) participating in TGP for face-to-face (*N* = 51) and online (*N* = 53) groups. GHQ-12 sum score ranging from 0 to 36; higher scores indicate a higher likelihood of potential mental health issues.

To investigate whether the face-to-face and online setting produce an effect on mental health, a repeated-measure ANOVA on mental health with setting (face-to-face vs. online) as independent variable was calculated. Here, a significant main effect for time and no interaction effect for time x setting was found, *F*(1, 96) = 33.06, *p* < .001, *η_p_*^2^ = .25 and *F*(1, 96) = .01, *p* = .93, *η_p_*^2^ = .00, respectively (see Figure 3) (for means and standard division per group, see Table 2). However, when controlling for gender, age, leadership role, teaching load, school year and repetition, analyses showed neither a significant main effect for time nor an interaction effect for time x setting, *F*(1, 96) = 1.81, *p* = .18, *η_p_*^2^ = .02 and *F*(1, 96) = .00, *p* = .97, *η_p_*^2^ = .00, respectively. Two control variables seem relevant for the effectiveness of the group program. First, for participation, the interaction with mental health over time approached significance: *F*(1, 96) = 4.45, *p* < .05, *η_p_*^2^ = .04. Mean score comparisons showed that participants who attended the training program for the first time showed a greater decrease in mental strain than participants who attended repeatedly (mean_t1_ = 15.58 (5.55), mean_t2_ = 10.74 (4.91) vs. mean_t1_ = 14.12 (5.47), mean_t2_ = 12.53 (5.85)). Secondly, there was a tendency for an interaction effect between leadership role and mental health over time, *F*(1, 96) = 3.74, *p* = .056, *η_p_*^2^ = .04. *post-hoc* results revealed that participants without a leadership role benefited more from the attendance of the program than participants with a leadership role (no role: mean_t1_ = 15.30 (5.68), mean_t2_ = 11.09 (5.33) vs. role: mean_t1_ = 14.05 (4.96), mean_t2_ = 12.64 (5.25)).

**Table 2:** Pre-post mean scores and standard deviation of dependent variables for both groups.

	pre-test		*t*-test	post-test		effect size *η_p_*^2^
	face-to-face group	online group		face-to-face group	online group	
Mental health^a^	15.06 (6.27)	15.02 (4.80)	.04	11.49 (5.90)	11.34 (4.78)	.00
General life satisfaction^b^	3.60 (.78)	3.98 (.60)	−2.77**	3.80 (.77)	4.03 (.61)	.02
Ability to distance oneself^b^	2.80 (.73)	2.71 (0.76)	.56	2.93 (.73)	2.90 (.84)	.00

** *p* < .01.

^a^ measured by GHQ-12 sum Score, ranging from 0 to 36, higher scores indicate a higher likelihood of potential mental health issues.

^b^ AVEM mean score, range from “strongly disagree” (1) to “strongly agree” (5), higher scores indicate a more pronounced tendency of the respective dimensions.

Concerning general life satisfaction, results from the repeated-measure ANCOVA showed neither a significant main effect for time nor an interaction effect time x setting, *F*(1, 96) = .41, *p* = .52, *η_p_*^2^ = .00 and *F*(1, 96) = 1.78, *p* = .19, *η_p_*^2^ = .02, respectively.

For the ability to distance oneself, repeated-measure ANCOVA revealed a main effect over time, but no interaction effect time x setting, *F*(1, 96) = 4.31, *p* < .05, *η_p_*^2^ = .04 and *F*(1, 96) = .04, *p* = .85, *η_p_*^2^ = .00, respectively. Overall, participants in both groups showed better ability to distance themselves after the group program (M_t1_ = 2.75 SD = .74, M_t2_ = 2.91 SD = .79). Again, an interaction effect for the control variables repetition as well as for leadership role was found [*F*(1, 96) = 6.95, *p* < .05, *η_p_*^2^ = .07 and *F*(1, 96) = 3.57, *p* < .05, *η_p_*^2^ = .04].

For means and standard divisions per group of the dependent variables, see Table 2.

## 4 Discussion

The present study aimed to evaluate an established group-coaching program (TGP) to promote teachers' mental health in two settings: face-to-face and online. Originally designed in the face-to-face setting, since the outbreak of COVID-19, TGP is administered online as well. To investigate whether online TGP differed from the face-to-face setting, the study compared satisfaction with and effectiveness of TGP between both settings. To our knowledge, this is the first time that a group-coaching program targeting teachers' mental health in the face-to-face setting and the online setting has been studied in detail. Specifically, this is one of the first studies investigating the Balint group technique in an online setting. The findings provide valuable insights into the feasibility and effectiveness of online mental health interventions for teachers and the translation of face-to-face group-coaching and the Balint group technique in the online setting.

### 4.1 Participants showed high satisfaction level with TGP in both settings

The evaluation during TGP showed that participants in the online setting reported a high satisfaction with the content of the intervention, its personal value and the moderator (good to very good). Satisfaction was particularly high at the end of the intervention (session 6). This aligns with Lodder et al. (80) who also found that participants are positive about videoconferencing for a group support intervention. Nevertheless, face-to-face participants evaluated all items (the relevance of topics, intra-group communication, learning experience, relief, moderation, and perceived personal value) even better during the intervention. Two possible explanations can be proposed. First, the moderators of online groups may still lack experience in reaching the same level of quality as those of the face-to-face groups, which have existed for over ten years. In the face-to-face groups, TGP satisfaction levels rose over the years, and there might be a ceiling effect in these groups (see e.g., Lahmann et al.^1^). Secondly, due to the online environment, bonding and trust, as essential elements of the Balint technique (81), might not develop as easily and quickly as in the face-to-face setting. However, satisfaction in key areas of the TGP (e.g., the relevance of topics, intra-group communication) was still high and very acceptable in the online setting. Moreover, in the retrospective post-test survey (two weeks after the last session), participants who attended at least five of the six sessions showed the same level of satisfaction for online participation as in the face-to-face group, and even higher satisfaction levels with regard to moderation. This indicates that although it might take longer to develop, trustful relationships as well as reciprocal learning can be established in the online setting in a similar way as in the face-to-face setting. Key reasons for these results are the experience and psychotherapeutic background of our moderators. In line with our findings, other studies also showed that the quality of the of therapeutic alliance does not differ between the online and face-to-face settings (82, 83). In contrast (84), found in their study that face-to-face treatments are superior in this regard. However, therapeutic experience seems to play a key role in the online setting. Lin et al. (85) found in their meta-analysis that trainee therapists had higher client attrition rates than licensed therapists in videoconferencing therapy. These results underline the importance of experience and using trained professionals for online therapy and—in the context of the current study—TGP, particularly for the online setting.

In sum, both groups showed high satisfaction levels across all three measurements. Even though there were some differences between satisfaction levels of the face-to-face and online group during the TGP process. In the end, both groups showed similar results and the online group showed even higher satisfaction rates at T2 after TGP was finished. As a longitudinal within-subject comparison was not possible due to the anonymity of the procedure, future studies should focus on finding a way to retrieve this data for better intra-person comparison and to evaluate whether there are differences during the coaching group- or therapy-process, for example in therapeutic alliance.

### 4.2 Participants showed improved mental health in the face-to-face as well as the online setting

Several aspects need to be discussed when regarding the influence of TGP on mental health and work-related attitudes. First, participants showed a decrease in mental distress over time in both settings. This is in line with previous findings that showed improved mental health of participating teachers compared to a non-contact control group (68–71). Thus, together with the previous body of literature, the current study supports the assumption that TGP has a relieving effect on teachers' mental health distress.

Second and most importantly for the scope of the paper, our study did not find any interaction effect between time (pre-post-test changes) and setting in relation to mental health. This result suggests that both delivery methods lead to enhanced teachers' mental health through TGP. This critical finding underlines the potential of online interventions as an effective alternative to traditional face-to-face programs, especially when in-person meetings are not feasible, e.g., during the COVID-19 pandemic or in rural areas due to distance. Our results are compatible with findings in the area of psychotherapy. Earlier studies found no differences in relation to post-treatment outcomes between in-person face-to-face therapy and online therapy for mood and/or anxiety disorders [e.g., (21, 86)]. A meta-analysis synthesizing results from RCTs comparing tele-therapy (telephone and videoconferencing therapy) to face-to-face therapy found no difference in treatment outcomes at post-test and follow-up between both settings (85). Although psychotherapeutic RCT studies yield similar results, it is hard to conceptualize the current study results in this context. The current study is a prevention program that applies a group coaching setting with “healthy” teachers, which is vitally different to one-on-one therapy with diagnosed clients. Unfortunately, to our knowledge, no published studies that investigate group prevention programs in the work context applied in both settings. Even though online interventions (in a therapeutic context as well as in the work setting) seem to be effective in reaching relief and behavioral change, more studies are needed that directly evaluate possible mechanisms behind the effects of online coaching (e.g., expertise of moderator, group setting, topics).

Third, no difference was found between the face-to-face and online groups in relation to work-related attitudes. However, the study found two different outcomes for general life satisfaction and the ability to distance oneself from work. On the one hand, results revealed no pre-post- or interaction effect for general life satisfaction. On the other hand, the ability to distance oneself from work showed improvement over time for both face-to-face and online groups. Hereinafter, both results are discussed separately. General satisfaction with life (as measured in this study) as an overall scale for well-being seems to change in the positive as other factors shift in the desired direction: Braeunig et al. (68) have shown that in those participants of TGP for which mental health has improved significantly, work-related factors are positively correlated. As a result, general satisfaction with life also increases. Thus, general life satisfaction seems to be more global well-being scale, it might not be as sensitive to change as more specific scales such as mental health or the ability to distance oneself from work. Therefore, it is not surprising that TGP does not significantly improve this scale for all participants. In addition, mean scores are rather high in the current sample making enhancement or change difficult. Future research in a larger sample is necessary to evaluate whether TGP online and face-to-face could lead to changes in general life satisfaction. Specifically, extreme group evaluation could be suggested to detect changes in participants with low scores in general life satisfaction. The pre-posttest improvement for the ability to distance oneself from work is in line with a previous study that showed this ability as a key factor acquired through TGP (see 68). The Balint group technique specifically helps to distance oneself (physically and emotionally) from the situation by leaving the group circle after the scenario was described. Distancing has been demonstrated as a key component in emotion regulation (87), which in turn is highly correlated with employee mental health particularly in professions where emotional labor is a main task (such as teaching) (88–90). It is important to note that there was no difference between TGP face-to-face and TGP online, suggesting that this key aspect for emotion regulation is learned in both settings. Interestingly, even though online the participant cannot remove her/himself physically from the group cycle, the effect is achieved. This finding again supports that trained moderators can transfer key elements of TGP into the online setting and similar desirable intervention effects can be reached. It would be interesting to investigate if these findings can be confirmed to different working populations such as doctors or professional care takers as key targets of the Balint group technique.

Taken together, the absence of intergroup differences in relation to the dependent variables suggests that both delivery methods do not differ significantly in enhancing teachers' mental health and ability to distance themselves from work in the current sample. Although, the absence of an effect is not the evidence of equality of the groups, looking at the mean scores, *p*-values, the absence of effect(-size)s and the self-evident very parallel changes from T1 to T2, strong similarity between the effectiveness of the interventions could be proposed for the current sample. However, future studies with a larger sample need to test this hypothesis further with a non-inferiority or equivalence approach.

Lastly, two co-variables showed significant influence on the effectiveness of TGP: leadership role and repeated participation. School principals could possibly benefit less from the intervention, as TGP primary targets teaching inherent challenges. The day-to-day work of teachers consists primarily of teaching and interacting with the students, their parents or colleagues. In comparison, school principals spent significantly less time teaching and more time with administrative and organizational tasks, which might limit their access to the potential benefits of this intervention. However, future research should investigate whether school principals would benefit from different modules. The analyses showed furthermore that participants attending for the second time did not benefit from the program to the same extent as first-time participants. This is not surprising as the repeated participation group showed better mental health at T1 than participants attending for the first time. This is consistent with an unpublished study by our group that found reduced, but still present, positive health effects 6 and 18 months after the last session. Nevertheless, repeated participation in TGP still leads to less mental distress. This suggests that repetition leads to stabilization of the learned skills and attitudes that promote successful relationships and in turn lead to the associated positive health effects. In sum, those newcomers with no leadership role who participate for the first time seem to benefit most from TGP, regardless of the setting.

### 4.3 Limitations and future research

When generalizing and interpreting the results, some limitations need to be considered.

First, due to the nature of the intervention program (public health care) a randomized RCT study was not possible. However, neither intervention group differed in regard to gender, teaching load, leadership role, school type and mental health at T1. Without including a control group, assessing the effectiveness of either setting might seem questionable. However, previous studies (69–71) already included non-contact control groups and found similar results, so we are confident that our results can be generalized in this direction.

Second, we reduced the analyzed dataset under very strict criteria and therefore had a large dropout of participants: about 42% of participants were excluded due to incomplete data at time T1, avoiding to deal with missing values. While this high exclusion rate might suggest issues with our data collection procedures or participant commitment, potentially limiting the representativeness of the findings, it ensures that the analysis is based on reliable and complete core data, enhancing the quality of the research findings. Furthermore, a significant number of participants were excluded because their data could not be matched between T1 and T2, affecting over half of the initially registered participants. This substantial reduction in sample size could introduce a bias and affect the study's power. However, the matching process is crucial for the integrity of longitudinal research as it ensures that conclusions are based on consistent data across two points. The study also excluded participants who attended less than five sessions, potentially skewing the sample towards more engaged individuals. This exclusion could bias the results as it might not reflect the outcomes of a differently engaged audience. On the other hand, our consistent approach helps focus the analysis on the impact of the intervention under optimal conditions (dose) where participant engagement and “treatment” is sufficient to fully test the intervention's effectiveness [see previous studies (70, 71)]. However, an intent-to-treat approach could be applied in future studies to increase the sample size (power) and investigate the optimal dose of TGP needed for a successful mental health improvement by participating teachers. Finally, the drastic reduction to the remaining 104 participants in this study, dividing almost evenly between face-to-face and online groups, could significantly limit the statistical power. Still, this balanced approach allows for a controlled comparison between two delivery modes of the intervention providing valuable insight into how different settings might affect participant outcomes. In addition, the sensitivity analysis revealed that, with a total sample size of 104 participants, the smallest detectable effect size is *f* = 0.138 while maintaining a statistical power of *β* = 0.80. This indicates that the sample size is sufficient to detect small to medium effects, ensuring that the study is adequately powered to identify meaningful differences or interactions, if they exist. As a result, the sample size does not pose a limitation for the detection of relevant effects in this study. Moreover, there was no significant difference in age, gender, teaching load, leadership role and school type between the study sample and the excluded sample at T1 (see Supplementary Table S1).

Third, reliance on self-reported measures for assessing mental health is a limitation. Self-report data can be subjective and prone to biases such as social desirability or inaccurate self-assessment. To enhance the robustness of findings, future studies could incorporate more objective criteria such as biomarkers for stress. However, third-party opinion (from moderators) is collected at the end of each academic year.

To address the limitations identified in this study, future research should include a non-contact control group to enhance the external validity of the research and complement self-reported measures of mental health with more objective measurements such as records of sick leave and biological stress markers (e.g., hair cortisol etc.).

### 4.4 Practical implications

For schools and policymakers, these findings highlight the feasibility of implementing online mental health support programs as part of comprehensive teacher well-being initiatives. Particularly important for psychotherapy research, the present study points towards the possibility of using established face-to-face methods in the online context yielding to similar and satisfying results. Given the logistical challenges and resource restraints often associated with face-to-face programs, online group coaching offers a scalable and flexible alternative that can reach a broader audience or appeal to persons who cannot be reached by a time intense face-to-face program. However, as this is the first study investigating Balint group technique in the online setting, there is more research needed to investigate whether all participants benefit the same from the online format (e.g., school leaders). As TGP and other therapy programs have proven to be effective over various years in the face-to-face setting, the online setting might be a good add-on to reach a specific more (skeptical) subgroup. However, the future might bring more hybrid models, which combine online and in-person elements. These could be particularly effective in maximizing both accessibility and participant satisfaction.

## Data Availability

The raw data supporting the conclusions of this article will be made available by the authors, without undue reservation.

## Footnotes

^1^Lahmann C, Lütke Lanfer SS, Pfeifer R, Rieder Y. *Ergebnisse aus der Begleitevaluation des Präventionsprojekts “Lehrer*innen-Coachinggruppen nach dem Freiburger Modell” [Project report “Teacher-Coaching-Groups“].* University of Freiburg, Germany, unpublished manuscript (2023).
