# Supplementary material for: ONLINE vs. FACE-TO-FACE group coaching to promote teachers mental health: an exploratory field study in German teachers
Source: Front Digit Health. 2025 Feb 5;7:1479524. doi: 10.3389/fdgth.2025.1479524 (PMC11836030; doi:10.3389/fdgth.2025.1479524)
Supplement: Supplementary file 1 [file Datasheet1.docx]

Supplementary Material

# Supplementary Tables

**Supplementary Table S.1.** Demographics teaching and training related variables for the study sample versus excluded participants.

|  |  | Study sample | | | | | Drop-out | | | | | | χ²-test p | | | |
| --- | --- | --- | --- | --- | --- | --- | --- | --- | --- | --- | --- | --- | --- | --- | --- | --- |
|  | *N* | 104 | | | | | 395 | | | | | |  | | | |
| *Demographics* | |  | | | | |  | | | | | |  | | | |
|  | **Gender**  female  male | 94  10 | | | (90.4%)  (9.6%) | | | 345  49 | | (87.6%)  (12.4%) | | | | .428 | |  |
|  | **Age (years)**  < 35  35-39  40-44  45-49  50-54  >55 | 12  9  13  19  25  26 | | | (11.5%)  (8.7%)  (12.5%)  (18.3%)  (24.0%)  (25.0%) | | | 65  48  81  60  69  72 | | (16.5%)  (12.2%)  (20.5%)  (15.2%)  (17.5%)  (18.2%) | | | | .099 | |  |
|  | **Teaching load**  100%  >75%  50-75%  <50% | 39  27  34  4 | | | (37.5%)  (26.0%)  (32.7%)  (3.8%) | | | 171  107  91  26 | | (43.3%)  (27.1%)  (23.0%)  (6.6%) | | | | .188 | |  |
|  | **Leadership role**  No  Yes | 82  22 | | | (78.8%)  (21.2%) | | | 325  70 | | (82.3%)  (17.7%) | | | | .422 | |  |
| *School type* | |  | |  | | |  | | | | |  | | .506 | |  |
|  | Basic elementary school (1^st^- 4^th^ year) | 26 | | | (25.0%) | | | 101 | | | (25.6%) | | |  | |  |
|  | Secondary school (5^th^- 9^th^ year) | 3 | | | (2.9%) | | | 18 | | | (4.6%) | | |  | |  |
|  | Secondary school (5^th^- 10^th^ year) | 10 | | | (9.6%) | | | 46 | | (11.6%) | | | |  |  |  |
|  | High school (5^th^-13^th^ year) | 24 | | | (23.1%) | | | 88 | | (22.3%) | | | |  |  |  |
|  | Community school | 4 | | | (3.8%) | | | 25 | | (6.3%) | | | |  |  |  |
|  | Vocational school | 17 | | | (16.3%) | | | 58 | | (14.7%) | | | |  |  |  |
|  | Special schools for mentally or physically handicapped pupils | 19 | | | (18.3%) | | | 59 | | (14.9%) | | | |  |  |  |
| *Training related variables* | | |  | | |  | | |  |  | | | |  | |  |
|  | **Repeated participation**  No  Yes | | 56  48 | | | (53.8%)  (46.2%) | | | 292  103 | (73.9%)  (26.1%) | | | | <.001 | |  |

*Note*: *N* = 499; frequency (percentage in group); χ²-test *p* between groups

**Supplementary Table S.2.** Summary of intervention manual

| Module | Description |
| --- | --- |
| Effects of relationship experiences on health: neurobiological basics | This coaching module offers an introduction that explores the links between interpersonal relationships and physical health. Using insights from neurobiology, the module illustrates how relational experiences can significantly affect neurobiological and their physical health. Neurobiological evidence shows that relationship experiences influence physiological processes in the body. Chronic stress, exclusion and humiliation activate the stress axis and have long-term consequences, such as sickness and mental health problems. By contrast, affection, recognition and attention deactivate this axis and activate the motivational systems instead. |
| Personal attitudes: identity and identification | In this module, participants address the question of how to find and keep a balance between their own identity and their professional role as a teacher. Being able to express one's own personality is a fundamental source of strength and resilience. It makes us authentic and genuine in contact, but it can also make us vulnerable in the school environment. Taking on the professional role as a teacher offers protection, whereby "too much" can lead to distancing or over-identification and perfectionism. The aim should be to achieve a healthy expression of one's own identity and emotions while at the same time maintaining a professional attitude and boundaries. |
| Shaping relationships with pupils | This coaching module is centered on developing effective relationships with students by understanding and balancing the elements of "empathy" and “leadership” in the educational context. For a good relationship, it is important to understand the students and to recognize them as individuals. This is vital to be able to share common goals and attention. Additionally, it emphasizes the crucial component of "leadership" in teaching, which involves setting and maintaining clear expectations regarding social rules, values, and achievable academic goals. |
| Building relationships with parents | This coaching module addresses the often-challenging relationship between teachers and parents of their students. It emphasizes the need to better understand and improve these interactions. The module discusses the mutual distrust and negative assumptions that frequently characterize the relationship between teachers and parents, urging participants to explore and clarify these dynamics. Contact with parents can be an opportunity to remember the common goal and strengthen the role and task of the teacher in the classroom. |
| Strengthening collegiality and social support among the staff: conflict-ridden tendencies versus collegial cohesion | Positive relationship experiences with colleagues can deactivate the stress axis. Social support - especially from colleagues - promotes health. However, there is also a risk of many small divisions within the teaching staff (e.g. due to complaints from pupils/parents or more liberal didactic approaches vs. stricter ones, etc.). Raising awareness of these potential divisions and open communication can prevent disputes within the teaching staff and promote cohesion and mutual support. |
